# Diversity- and culturally sensitive approaches in nutrition and fluid management in long-term nursing. A scoping review

**DOI:** 10.1186/s41043-026-01346-2

**Published:** 2026-05-21

**Authors:** André Heitmann-Möller, Martina Hasseler, Michael Feldhaus, Monika Schlegel, Sandra Hellmers, Andreas Hein, Steffen Busse, Julia Berndt, Mareike Förster, Rebecca Diekmann, Tobias Krahn

**Affiliations:** 1 Ostfalia University for Applied Science, Faculty of Health and Health Care Sciences, Wolfsburg, Germany; 2 Carl von Ossietzky Universität Oldenburg, Department of Social Sciences, Sociology of the Life Course and Social Inequality, Oldenburg, Germany; 3 Carl von Ossietzky Universität Oldenburg, Department of Health Services Research, Assistance Systems and Medical Device Technology, Oldenburg, Germany; 4 OFFIS – Institute for Information Technology, Oldenburg, Germany

**Keywords:** Cultural sensitivity, Diversity, Migration background, Long-term care, Long-term nutritional intake, Fluid intake, Hydration, Framework

## Abstract

**Background:**

As a result of decades of migration in Germany, the number of persons with migration backgrounds from different cultures in residential long-term care nursing facilities will increase. As a result, cultural diversity may also have an impact on nutritional aspects in long-term care. The project ‘Digitally supported diversity and culturally sensitive nursing care on nutritional intake’ (NUTRI-SENSE) examines strategies to improve the nutritional and fluid intake of residents with migration backgrounds in long-term care facilities. The interdisciplinary project aims to improve their health and quality of life with a digitally supported nursing process to prevent undernutrition and dehydration. To synthesize the evidence on diversity- and culturally sensitive approaches in nutrition and fluid intake management, a scoping review was conducted. This research led to the question of the extent to which cultural sensitivity with regard to nutrition and fluid intake is addressed in long-term care nursing homes. A literature search of different databases (PubMed, CINAHL, LIVIVO, CareLit^®^, manual search: Google Scholar) was conducted in May and June 2025.

**Results:**

From the 8.010 findings, 28 publications were screened, and 6 publications were included in the review. The evidence on diversity- and culturally sensitive approaches in institutional long-term care nursing regarding nutrition and fluid intake is limited. The main topics are the relationship of culture-specific and dementia-specific needs; the emotional aspects of belonging, food and memories of residents with dementia; meals as a vital source of well-being in nursing homes; the meaning of mealtime experiences in a multicultural society; and, finally, the involvement of family members in the food supply.

**Conclusion:**

The findings from the scoping review revealed that a systematically developed, diverse and culturally sensitive framework for managing residents’ nutritional and fluid intake in long-term care facilities has not yet been established. Such a framework is the goal for subsequent research and the development of interventions within the NUTRI-SENSE project. In the context of international population ageing and increasing needs in the long-term care sector, the development and evaluation of culturally and diversity-sensitive nutPrition and hydration strategies are of broad, cross-national relevance.

**Supplementary Information:**

The online version contains supplementary material available at 10.1186/s41043-026-01346-2.

## Background

The four-year project ‘Digitally supported diversity and culturally sensitive nursing care on nutritional intake’ (NUTRI-SENSE), launched in May 2025 as a transdisciplinary consortium in Lower Saxony (Germany), examines strategies to improve residents’ nutritional and fluid intake in long-term care (LTC) while explicitly accounting for diversity and cultural sensitivity. Research activities comprise semistructured interviews with residents, family caregivers, registered nurses, nursing assistants, and managers in LTC facilities, complemented by ethnographic observations. A parallel workstream entails the development and evaluation of digital applications designed to support diverse and culturally sensitive nursing processes related to nutrition and fluid intake. The primary objective is to improve residents’ health and quality of life, especially for individuals with migration backgrounds. A scoping review was conducted at the beginning of the project to synthesize the evidence on diversity- and culturally sensitive approaches in LTC nursing, with particular attention given to nutrition and hydration management.

## Migration to Germany: Increasing diversity among residents of long-term care homes

As a result of decades of migration to Germany, the number of people with a migration background who require nursing care can be expected to rise (Olbermann 2020). The reasons for migration are heterogeneous and shape migrants’ biographical characteristics. In addition, unfavourable living conditions in Germany, e.g., housing, employment, and educational opportunities, affect migrants, who later require long-term care [1]. Because of their long history of migration to Germany, migrants represent a highly heterogeneous group [2]. A recent statistical survey identified the largest groups with a migration background as originating from Turkey, Poland, the Russian Federation (ethnic German repatriates), and Italy [3]. In 2022, 23.8 million people and 28.7% of the total population were migrating, whereas in 2021, 22.6 million (27.5%) were migrating. The number of foreign nationals rose from 10.6 million in 2021 to 11.6 million in 2022 (+ 9.7%) [3]. Owing to demographic changes and reduced family solidarity, the potential for care support from younger family members in migrant families is expected to decline, increasing the need for professional nursing care [4].

Residents with migration biographies often experience language limitations [5]. Empirical evidence indicates that nursing staff may not adequately identify needs and culturally specific preferences related to nutrition, personal hygiene, or social activities [5]. Nursing homes therefore need to adapt to culturally and diversity-sensitive care [5]. Culturally sensitive nursing care aims to enable people in need of care to live according to their individual values and cultural and religious imprints or needs [6]. It entails reflecting on norms, rituals, ceremonies, and feelings such as shame or embarrassment that affect both caregivers and those receiving care [7]. This approach is limited by the heterogeneity of older adults, particularly with respect to social inequality and health status, which is shaped by the diversity of social milieus in an ageing German society [8]. Moreover, a growing pluralization of lifestyles and changes in sociocultural values among older people can be observed [8, 9], which extended the diversity of this population. Expectations concerning long-term care among people with migration backgrounds may also differ from those of the autochthonous population [1]. Differentiated long-term care concepts that meet the needs, requirements, and expectations of people with a migration background have yet to be developed [1], but long-term care must also be organized in a more efficient and less time-consuming manner. On this background the terms culturally-sensitive and diversity-sensitive could be defined as core aspects of an individual- respective person-centred nursing practice in long-term care, which entails the consideration of extra- and intra-individual factors.

## Meaning of nutrition and fluid intake in long-term care

Good nutrition and adequate nutritional status are integral to health, independence, and quality of life in older people [10, 11]. Regular monitoring of nutritional and fluid status is essential, particularly for older people with a migration background, disabilities, or other limitations [12–14]. The ESPEN guidelines on nutrition and hydration in geriatrics recommend standardized procedures, beginning with routine screening followed by systematic assessment [12]. Nutrition is central to culturally sensitive care because malnutrition adversely affects the development of long-term care needs, health outcomes, and subjective well-being [15, 16]. Inadequate nutrition and fluid intake in older people with healthcare needs affect immune function, cognition, and mobility; they increase the risk of infections, delayed wound healing, falls, delirium, altered drug metabolism, and deterioration of physical and cognitive function [12, 17]. Additional risks include decreased muscle strength and consequent falls, skin and tissue damage, pressure ulcers, neurological and cognitive impairment, renal and cardiological complications, and reduced quality of life [14]. In Germany, evidence indicates that nursing home residents with a migration background are more often undernourished than German residents are [18]. These consequences heighten vulnerability in long-term care [15, 19, 20].

There is broad consensus in nursing research that a biosocial approach to nutrition in long-term care is needed [21]. This approach should integrate evidence-based nutritional recommendations with medical histories and eating biographies to promote ageing with dignity and optimal health [21]. The nursing process provides an appropriate implementation framework: as a central tool for organizing nursing practice, knowledge, and care, it aims to realize person-centered, needs-based care [22, 23]. The nursing process comprises an iterative sequence of assessment and diagnosis, nursing care planning, implementation, and evaluation. It should be performed regularly and documented in a standardized nursing language. However, heavy workloads often hinder systematic application.

### Question of diversity- and culture-sensitive approaches in long-term nursing care

The implementation of diversity-sensitive nursing care/long-term care and nutrition and fluid intake depends on multiple factors, including personal and environmental determinants [24]. Given increasingly heterogeneous nursing home populations, nurses and care assistants must recognize each person as an individual with biographical experiences and needs, as well as as familial, social, cultural, and spiritual, shaped by age, gender, and experiences of suffering [25]. These aspects influence nutrition and fluid intake among residents with migration backgrounds and are crucial for well-being, maintenance of function, autonomy, and quality of life [12]. In Germany, diversity- and culture-sensitive approaches to nutrition and fluid intake in long-term nursing care have not yet been examined. This leads to the question of which approaches exist in long-term care, especially in nursing homes, with respect to nutrition and fluid intake. To address this, we conducted a scoping review to explore this topic.

## Method

This scoping review was undertaken within the NUTRI-SENSE project to research the following question:

To what extent is the issue of cultural sensitivity with regard to nutrition and fluid intake addressed in nursing homes?

Scoping reviews generally include all scientific evidence and publication types relevant to the research question without assessing methodological quality [26]. Nevertheless, scoping reviews may assess the quality of reporting and risk of bias in reviews and observational studies via established instruments [26, 27]. We draw on the Preferred Reporting Items for Systematic reviews and Meta-Analyses extension for Scoping Reviews (PRISMA-ScR) for writing the report [28]: First, study characteristics and indications of bias are presented, followed by a detailed description of each included study. Third, a synthesis of the findings is provided for each research question.

### - Search strategy -

For the literature searches, English search strings were formed to direct the search in scientific databases according to the above mentioned research question. Regarding three search terms corresponding MeSH-terms were included in the search string: The term “diversity sensitive” corresponds the MeSH-term ‘cultural diversity’, the term ‘cultural sensitive’ corresponds the MeSH-term ‘culturally competent care’ and at last the term ‘nutrition’ corresponds the MeSH-term ‘nutritional status’. For ‘long-term care’, ‘nursing home’ and ‘nursing process’ the identical MeSH-terms were used. Regarding the ‘nursing process’ additional the acronym ‘ADPIE’ as an alternative term was used [29, 30]. No corresponding MeSH-terms for ‘hydration’ and ‘fluid management’ were found, in consequence the defined terms were used.

*(cultural diversity) OR (diversity-sensitive care)) AND (culturally competent care)) OR (cultural-sensitive care)) AND (Long-term Care)) AND (Nursing homes)) AND (Nutritional Status)) OR (Nutrition)) AND (hydration) OR (fluid management) AND (Nursing)) AND (Nursing Process) OR (ADPIE) AND (Assessment) OR (Diagnosis) OR (Planning) OR (Implementation) OR (Evaluation)*.

The above mentioned search string was used in PubMed, but according to more restricted search options (e.g., limited number of Boolean operators in CINAHL) in the CINAHL, CareLit^®^ and LIVIVO databases, the combination of search terms was adjusted (e.g. regarding the German LIVIVO database several different combinations were used to narrow the search). A targeted search for a relevant publication was conducted in Google Scholar, using DOI and authors names, after all publications from the visited databases had been uploaded to CITAVI and subjected to pre-screening, without the publication known to the research team appearing among the search results.

CINAHL: *(cultural diversity) OR (culturally competent nursing care) AND (long-term care) AND (nursing homes) AND (nutritional status)*CareLit^®^: *(Cultural diversity) AND (long-term care)*.

LIVIVO: * 1 st search attempt: (diversity-sensitive care) AND (long-term care) AND (nursing home); 2nd search attempt: (culturally competent care) AND (long-term care) AND (nursing home); 3rd search attempt: (culturally competent care) AND (nutrition) AND (long-term care); 4th search attempt: (cultural sensitive care) AND (nutrition) AND (nursing home)*,* 5th search attempt: (cultural diversity) AND (nursing process); (culture) AND (nursing process) AND (nutrition)*.

Google Scholar: DOI 10.1108/IJMHSC-02-2019-0015; authors: Alain Girard, Asma El Mabchour.

### - Selection process and eligibility criteria -

The use of inclusion and exclusion criteria for study selection (Table 1) followed the pattern that inclusion and exclusion criteria determine which sources from the databases should be selected for inclusion in or exclusion from the scoping review. This applied to titles and abstracts in the determined selection of the sources. The selection of relevant publications was conducted in four steps in CITAVI with two team members as a review team involved. First, the keyword function of CITAVI was used to select relevant publications according to the search terms in the pre-screening phase. Second, the titles of the publications were read. Third, after the publications met the inclusion criteria, the publications were further included in the screening process. In this step, the abstracts were screened. After this, the eligible publications were read according to the inclusion and exclusion criteria. Uncertainties during the steps of the selection process were discussed by the review team and, if applicable discussed in the whole research team, after which the final inclusion or exclusion of publications occurred.

**Fig. 1:**
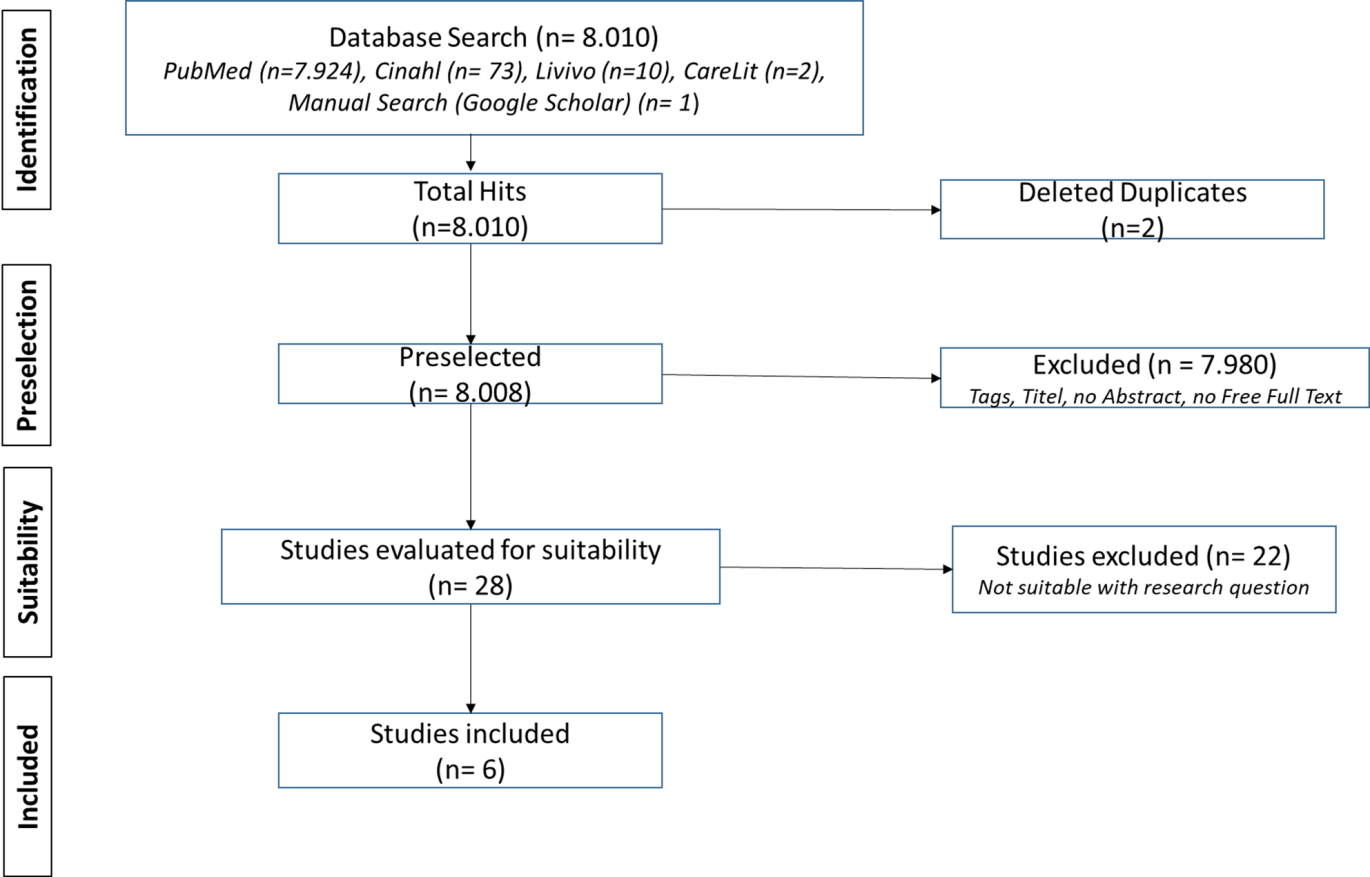
Selection process according the PRISMA statement

The two-month literature search of the databases was conducted in May and June 2025. Figure 1 visualizes the methodological procedure of the study selection process. The database search and manual search were divided between 2 researchers as reviewers to avoid overlaps and to ensure transparency. The literature search across the specified databases was conducted simultaneously at the beginning of the two-month period. During the subsequent phase of this period, the screening and selection of literature records were carried out. Citavi 7 was used as the common literature management program. A total of 8.010 publications were found, 2 duplicates were deleted, and 8.008 publications were screened. Finally, of the 28 publications evaluated for suitability, 6 studies were included. Two studies used secondary data from previous publications, and the other studies were based on original data from their own research projects. Oriented on PRISMA-ScR, the data extraction was performed by the reviewers and included the research questions or objectives, addressed topics or phenomena, methods, summaries of the core results and limitations or biases. The critical appraisal was conducted on a descriptive level according the above-mentioned extraction steps.

Publications in English and German from all countries, published between January 2015 and May 2025, were included in the search process. The publications should not focus solely on an English- or German-speaking cultural contexts, but should also take into account other cultural contexts. No study from the German-speaking context was identified. The publications examined originated from Australia, Canada, Malaysia, Sweden, Norway, and South Africa. Studies published in languages other than English or German were excluded. Additionally, studies focusing on unrelated topics, such as ambulatory nursing care, medical or specialized treatment approaches, and clinical studies related to long-term care in nursing homes, were also excluded. Studies with secondary data (e.g., literature reviews) were included. As a result, the publications stemmed from Australia, Canada, Malaysia, Sweden, Norway, and South Africa. Studies in a language other than English or German were excluded. Additionally, studies on various topics, including ambulatory nursing care, medical or specialist treatment approaches, and clinical studies related to long-term care in nursing homes, were excluded.

**Table 1:** Inclusion/exclusion criteria

Inclusion criteria	Exclusion criteria
• Reference to diversity- and culture-sensitive care concepts • Reference to diversity- and culture-sensitive nutrition and hydration • Reference to long-term care nursing homes • Freely accessible text • Publication language: English, German	• Text is not freely accessible • Reference to ambulatory nursing care • Medical or specialist therapeutic treatment approaches • Clinical studies

## Results

### Evidence on diverse and culturally sensitive approaches in institutional long-term nursing regarding nutrition and fluid intake

#### - Study characteristics -

The studies included in the scoping review are characterized by the following characteristics: Study 1: The investigation by Cabote et al. [31] is grounded in an integrative literature review, incorporating five distinct databases to comprehensively synthesize literature on the topic. Study 2: In their qualitative study, Xiao et al. [32] conducted interviews across four nursing homes in Australia, which included 16% residents from culturally and linguistically diverse (CALD) backgrounds. A semi structured interview guide was meticulously employed, enabling participation from both CALD and non-CALD residents as well as at least one family member for each interview. Study 3: The research conducted by Harstäde et al. [33] also utilized an integrative literature review methodology, engaging a range of diverse databases to gather relevant studies and findings. Study 4: The work of Hannssen and Kuven [34] comprised qualitative interviews utilizing a narrative approach, organized into three substudies. These substudies were executed in six residential facilities situated in South Africa and Norway, encompassing one home for Sami individuals and another for Norwegians. The study incorporated perspectives from nursing staff and family members during the interview process. Study 5: Chees’s investigation [35] adopted aphenomenologically oriented qualitative framework and conducted in-depth interviews to explore the lived experiences surrounding multicultural meals in long-term care facilities in Malaysia. A total of twenty-eight participants (14 women and 14 men) representing the Bumiputera Malay, Chinese, and Indian ethnic groups were involved. The researcher visited six long-term care facilities across three urban districts, justifying the use of a phenomenological approach based on Husserl’s philosophy to prioritize the direct experiences of the subjects over theoretical or interpretative heuristics. Study 6: This ethnographic study [36] combined structured nonparticipatory observations with semistructured interviews across three distinct social strata: residents of nursing homes who emigrated from Quebec 40 years ago (*n* = 26), their family members (*n* = 24), and nursing staff (*n* = 51) serving at both the assistant and specialist levels. Additionally, semistructured focus groups were conducted to enrich the qualitative data collected.

**Table:** Table Study Characteristics 2

Nr.	Objectives, topics and phenomena	Methods	Core results	Limitations/ biases
1	Examination of needs of older people with dementia from culturally and linguistically diverse backgrounds in residential aged care institutions.	integrative literature review	Culture-specific needs: • common language, • traditional food, • social and spiritual requirements Dementia-specific needs: • focusing on comfort in addition to clinical requirements • individualized care addressing behavioural symptoms	• no inclusion of evidence published in non-English journals • no settings-specific analyse, e.g. no distinction was made between ethno-specific facilities and mainstream institutions
2	Examination of residents’ and family members’ perceptions about staff and cultural diversity, culturally and linguistically diverse residents’ and family members’ experiences.	qualitative interview study	• perceiving diversity as an attraction • adapting to cross-cultural communication • adjusting to diet in the residential care home • anticipating individualized psychosocial interactions	• restriction to interviews as a key limitation • low willingness to participate among CALD (culturally and linguistically diverse) residents
3	Analysis of mealtime situations in nursing homes from the residents’ perspective.	integrative literature review	Mealtimes… • as a basis of wellbeing in nursing home life, • (re)creating continuity in life and preserving identity • as a harmonizing act between autonomy and need of support	• grey literature was overlooked • findings are country specific, transfer to other contexts must be done with caution • limited generalizability of findings in Western contexts regarding the global context
4	Investigation concerning the meaning of traditional food to patients with dementia in residential homes.	qualitative interview study	• dishes create positive recollection of childhood experiences, leading to maintain/strengthen cultural identity, create joy, feelings of belonging, respect and to be cared for	• study’s results cannot be generalized due to its qualitative design
5	Exploration of the lived experiences and meanings of mealtimes for older adults from multicultural backgrounds in senior living facilities in Malaysia.	phenomenologically oriented in-depth interviews	• mealtimes as cultural bridges, • memories and palate, • emotional bonds through food, • quality control and consumption, • comfort through personalized dining experience	• findings cannot be generalized due to its qualitative design
6	Better understanding of the meal context and the food offering in Quebec public nursing homes for nonautonomous seniors form the first-immigrants generation	ethnographic study	• difficult adaptation to food offering, often not at all offered food • food quality, mealtime schedules, medication intake, physical and mental condition, and adaptation to institutional life influence the resident’s appetite • supplementary food was provided by family/friends • because of political and organizational changes nursing care became more tedious, routinized with impacts on quality of care	• study aims solely to explore the perceptions and perspectives of the individuals involved regarding meals, meal service, food, recipes, the context of mealtime, institutional food offerings, and the manner in which food is presented

The significance of food intake within nursing homes emerges as a pivotal factor influencing the health and well-being of residents. Malnutrition, characterized by an insufficient intake of calories and essential nutrients, presents a formidable challenge for the elderly population residing in these facilities. Consequently, issues surrounding food frequently manifest as prevalent complaints among both residents and their families [37]. Furthermore, the experience of consuming meals transcends mere nutritional value, embodying a multifaceted social, identity-forming, symbolic, emotional, and hedonistic dimension. To grasp the complexities of food within this context, it is imperative to adopt sociocultural and anthropological approaches [36].

The critical importance of nutrition is especially important for individuals from migrant backgrounds, as the availability of culturally sensitive food options is markedly limited in most nursing homes. Despite its importance, the literature addressing diversity- and culture-sensitive nutrition within these institutions remains scarce. Existing studies predominantly explore the interrelation between culturally sensitive nutrition and the well-being of residents, as well as the potential avenues for its implementation in nursing home settings.

#### - Complementarity of cultural and dementia-specific needs -

A pertinent study conducted by Cabote et al. [31] included diverse participants, encompassing residents with dementia, family members, nursing staff, and managers, representing a wide array of cultural and ethnic backgrounds (e.g., African, Arab, Estonian, Filipino, Greek, Hungarian, Indian, Italian, Korean, Latvian, Malaysian, Swedish, and a smaller number from English-speaking countries). The authors delineated two primary themes in their findings: “culture-specific needs” and “dementia-specific needs.” Within the realm of culture-specific needs, several subthemes emerged. Notably, the need for a common language—more relevant between staff and residents than among residents themselves—was emphasized. Furthermore, traditional food was recognized as a tangible manifestation of support for residents’ cultural needs, serving as a means to honour their traditions. Family members, in particular, underscore the importance of this cultural sensitivity. The theme of “social and spiritual requirements” highlighted the experiences of isolation that residents with dementia may face in a culturally unfamiliar environment. Conversely, dementia-specific needs primarily encompass two subthemes: “comfort alongside clinical requirements,” which pertain to fostering comfort for residents despite the clinical nature of care, and “individualized care addressing behavioural symptoms of dementia.” The authors asserted that providing culturally appropriate food, employing suitable language, and respecting cultural practices could significantly enhance the care experience. Their conclusion posited that the needs of individuals with dementia from culturally and linguistically diverse (CALD) backgrounds predominantly align with culture-specific considerations, rendering dementia-specific needs of secondary importance. Nonetheless, it is essential to recognize that these needs are interrelated and should be addressed in an integrative manner.

#### - Potential of culturally familiar foods for CALD-

A subsequent publication explored the intertwined themes of belonging, food, and memories via interviews with family members of residents with dementia and experienced nursing staff [32]. The categorization revealed that traditional food enhances residents’ sense of identity and fosters social belonging within the nursing home environment. Additionally, the category labelled “food and memory” indicated that familiar aromas and tastes evoke joyful emotions linked to positive recollections of the past, possibly augmented by external stimuli such as the use of historical clothing, crockery, and cutlery. The calming effects of traditional dishes were noted, as they are often reminiscent of the residents’ formative years. Furthermore, the nursing staff reported an increase in appetite among residents when traditional dishes were served, underscoring the potential of culturally familiar foods to enhance overall well-being. Also, the authors identified “perceive diversity as an attraction” [32]. This refers to the fact that the residents interviewed and their families were aware of the cultural diversity of the staff in the facilities. They did not perceive this as problematic but rather as an attractive feature. This applied to residents with and without a CALD background. Xiao et al. [32] further emphasized the “adjusting process to diet in the residential care home”: Cultural background has a very strong influence on food preferences. Individuals with culturally and linguistically diverse backgrounds and their relatives prefer culturally specific dishes (e.g., people from East Asia prefer rice—prepared in the same way as it is at home). Moreover, nursing home residents are aware that not every meal prepared can meet their expectations. Here, families take on the task of providing their relatives with suitable meals. Overall, the authors believe that the topic of nutrition (or the selection of dishes/dietary options) appears to be the highest priority for CALD residents and their families.

#### - Meals as a vital source of well-being within nursing homes -

Harstäde et al. identified key themes in their integrative literature review, positing that meals constitute a vital source of well-being within nursing homes [33]. These meals not only fulfil nutritional needs but also provide crucial opportunities for residents to gather, fostering a sense of community and social belonging within the nursing home context. Despite the diverse nature of these facilities, the role of shared meals in promoting well-being and continuity of life remains profoundly significant. Catering and food intake in nursing homes are significant factors influencing the health and well-being of residents. Malnutrition, defined as a lack of food intake in terms of calories and nutrients, can become a primary problem for elderly residents. Catering is therefore one of the most common complaints of residents and their relatives [37]. However, food and meals are not only physically related to the health of residents. Eating meals also has strong social, identity-forming, symbolic, emotional, memory-related and hedonistic dimensions. For this reason, sociocultural and anthropological approaches are needed to understand food holistically [36]. The high importance of nutrition is particularly relevant for people with a migrant background, as the range of culturally sensitive food available is rather limited in most nursing homes. The topic of diversity- and culture-sensitive nutrition in nursing homes has rarely been addressed in the literature. The few studies that do exist address the connection between diversity- and culture-sensitive nutrition and the well-being of residents, as well as with opportunities for implementation in nursing homes [2].

#### - The essence of multicultural mealtime experiences -

Chee [35] identified several topics regarding the essence of multicultural mealtime experiences in senior living facilities. In the first topic, “Anchored in the past - mealtimes as cultural bridges,” Chee noted that family recipes, cooking techniques, and ingredients are passed down intergenerationally within the family, thereby establishing a genuine cultural tradition. The use of specific—culture-specific (Bumiputera Malays, Chinese, Indians)—ingredients is essential for maintaining this connection. Family gatherings with residents and shared meals at the facility serve as a bridge to connect the gap between life at the facility and past life in the family. This allows those affected to maintain their cultural roots by grounding themselves in their culinary family cultural history. The second topic, “Shared plates, shared stories - food feeds the heart and stomach,” highlights the importance of food as a means of maintaining relationships, especially under difficult conditions. Chee [35] refers here to the experiences of the interviewees during Japanese occupation in the second world war, when food supplies were severely restricted. At the same time, it becomes clear that food can also serve as a festive element of cultural occasions for maintaining relationships. Food serves here as a factor that generates and strengthens relationships, as well as maintaining and passing on meaningful memories. Topic 3, “The emotional and social significance of food—lingering traces of remembered affection,” revisits some interviewees’ memories of the aforementioned Japanese occupation. Solidarity within the family when sharing food plays a particularly important role here. Essentially, it becomes clear that food is not only a means of survival but also a source of comfort, hope, and resilience. Topic 4, “Consumption patterns and quality control—strengthening relationships, finesse, and texture of food,” refers to the relationship between residents and staff. The nature of this relationship can significantly influence consumption patterns and the quantity of food consumed. A positive relationship between them can foster an atmosphere of trust and comfort, which can lead to increased food intake. Conversely, a negative relationship can hinder adequate food intake. In particular, the texture of food can be an obstacle that affects its palatability and acceptance.

Culturally sensitive food in care homes increases the well-being of residents in several ways: it increases physical well-being by increasing the amount of food consumed. It supports the maintenance of one’s own traditions and identity. It increases social integration and promotes self-efficacy in food choices.

#### - Reasons for the involvement of families -

Girard and El Mabchour [36] identified several themes from interviews and focus groups that highlighted factors why families are involved in the food supply for residents with a CALD background. The first theme was the “meal context”: Each person received a personalized meal tray, whether in the dining room or in their private room. This was to ensure that they received the menu they had chosen and that dietary and health factors (e.g., by modifying the texture to facilitate food intake) were taken into account. The quality of the food varied greatly between facilities and depended on the experience and qualifications of the kitchen staff. Nursing assistants were involved mainly during mealtimes. Their responsibilities in the dining rooms included not only distributing food but also encouraging residents to eat their meals, ensuring that they eat enough, and making the process as safe as possible due to the risk of choking. The task was repetitive and demanding for the nursing assistants. Meal times in single rooms were presumably determined by family members or legal representatives or even by the residents themselves. This was due to various reasons: the state of health (e.g., advanced Parkinson’s disease or dementia), the shock of transition or difficulty to accustom oneself into the nursing home, lack of appetite, or lack of need for social contact. The inappropriate behaviour of fellow residents in the dining room due to their illnesses (e.g., dementia) also led to the decision to eat in their single rooms.

The researchers identified a second theme [36]: “Meals as an activity of sociability and maintaining a habitus.” Socializing was seen as an important purpose of meals, as being alone was not considered socially desirable. In particular, family members encouraged their relatives with dementia to participate in communal meals. At the same time, communal meals are believed to stimulate the appetite of people with a lower appetite through the sight of their fellow residents eating.

The third theme was “Assessment of food offerings and alternative practices of family members”, which reveals two groups of residents [36]: one group accepted the offer and emphasized that they were not picky. The other group was openly dissatisfied with the food offered. Only a minority of family members were satisfied with the food offered in nursing homes. In some cases, when there was dissatisfaction with the quality of preparation and cultural acceptance, food was brought from home or from restaurants. However, this had an ambivalent side: On the one hand, it was seen as a burden by relatives, but on the other hand, it also led to joint efforts by various unrelated families to bring food from home to the nursing home and thus support each other. Employees, especially nursing assistants, also considered the quality of the food in the nursing home to be unsatisfactory and perceived a deterioration compared with previous years. In addition, life before moving into the nursing home had probably led to the incorporation of an aesthetic of eating that was no longer taken into account in the institution. The reason for this lies in the requirements for food in shared accommodations.

#### - Limiting factors: Organizational conditions and politically driven management -

Previous findings also mentioned limiting factors for implementation processes: organizational conditions and politically driven management. In her study, Chee [35] identified the topic “Comfortable and welcoming dining environments” as an aspect related to the environmental factors of meals: dining rooms/dining areas are often very far removed from the comfort of a family home and therefore pose a significant challenge for residents. These settings embody an institutional atmosphere, marked by bare walls, minimal decor, and standardized seating configurations. From the interviews, Chee reported that some interviewees’ appetites and ability to enjoy food were limited by the sterile environment. Others experienced heightened feelings of isolation and loneliness, especially those who relied on meals as their only opportunity for social interaction. In addition to the functional, institutional character of dining rooms, there is a lack of personal touch and flexibility in accommodating individual meals. As a result, residents’ ability to organize a customized culinary experience for themselves would be limited.

Girard/El Mabchour [36] reported that the topic “Deterioration in the food supply and working conditions of nursing assistants” highlighted allegedly politically driven limits for culture- and diversity-sensitive nutrition. This situation was reported by nursing assistants and family members. Rising food prices, the loss of volunteers, organizational reforms that saved time in serving meals, and the loss of a culinary perspective due to budget cuts were blamed for this. Additionally, inflexible meal times had more to do with managerial and resource-saving imperatives than with the actual needs of the residents. At the same time, nursing assistants were pressured to adhere to standardized processes and were therefore no longer able to provide comprehensive support to those affected during mealtimes. The quality and preparation of food in terms of variety, taste, smell, and cooking technique are also considered barriers to eating. In this context, support and assistance with eating, either by family members, nursing assistants, or volunteers, seemed to be essential for preventing malnutrition.

#### - Challenges of provision of culturally sensitive nutrition -

In summary, providing culturally sensitive nutrition for older people in retirement homes can be challenging. Staff must initiate conversations to understand the preferences of older people, which can be very time-consuming but can also improve the psychological well-being of older people [35]. However, implementing culturally sensitive nutrition is very costly, particularly in terms of food quality, mealtimes, medication intake, physical and mental conditions, and adaptation to life in the facility, and requires a significant time commitment on the part of the nursing staff [36]. Therefore, offering traditional meals in nursing homes requires additional planning and resources, traditional knowledge, creativity, and knowledge of patients’ personal preferences [36].

## Discussion

The included studies were interdisciplinary: four involved nursing science, one originated from hotel/tourism management and nutrition science, and one from hotel/tourism management. Although the nursing process was included among the search terms, explicit references to it were uncommon in the retrieved articles. Two studies focused on dementia care. All studies used qualitative designs or literature reviews, with exploratory aims. The term “long-term care facility” varies across country-specific social systems. None of the articles addressed fluid intake explicitly, and most focused on nutrition. The lack of explicit mention of fluid supply or fluid intake in relation to culturally and diversity-sensitive long-term care therefore raises questions. This aspect is likely less prominent within this thematic field, and instead tends to be more prominently addressed in publications focusing on malnutrition and inadequate fluid intake in long-term care institutions. In consequence this topic has to be more investigated in future research. With respect to the research question, diversity- and culture-sensitive approaches in institutional long-term care appear to have been explored primarily in an exploratory manner regarding nutrition. Explicit concepts have yet to be explicitly published, perhaps reflecting the strong focus on nutrition.

The literature documents the widespread employment of care assistants in nursing homes. This raises questions about the consequences of delegating nutrition and fluid intake to personnel with lower formal qualifications and limited clinical training. International studies indicate that greater reliance on assistants relative to registered nurses is associated with lower quality-of-care indicators [38]. Budgetary constraints limit the recruitment of more highly educated personnel, prompting greater reliance on nursing assistants, who may be insufficiently trained to address residents’ autonomy and well-being; such skill-mix shifts may adversely affect care quality [39].

Nutrition and fluid intake are important quality indicators. In this context, a high proportion of care assistants are likely to influence not only nutrition and fluid intake in general but also culturally and diversity-sensitive nutrition and fluid intake.

The scoping review indicated that nutrition and fluid intake are not merely quality indicators or biomedical factors. They are also related to well-being, identity and sociality, which are shaped by culture. Despite the exploratory character of this scoping review the following indicators of culturally and diversity-sensitive approaches emerge: a primary focus on culture-specific influences on the well-being of residents as well as organizational conditions as significant limiting factors for culture- and diversity-specific nutrition (and likely fluid intake) in institutional long-term care [35, 36].

**Fig. 2:**
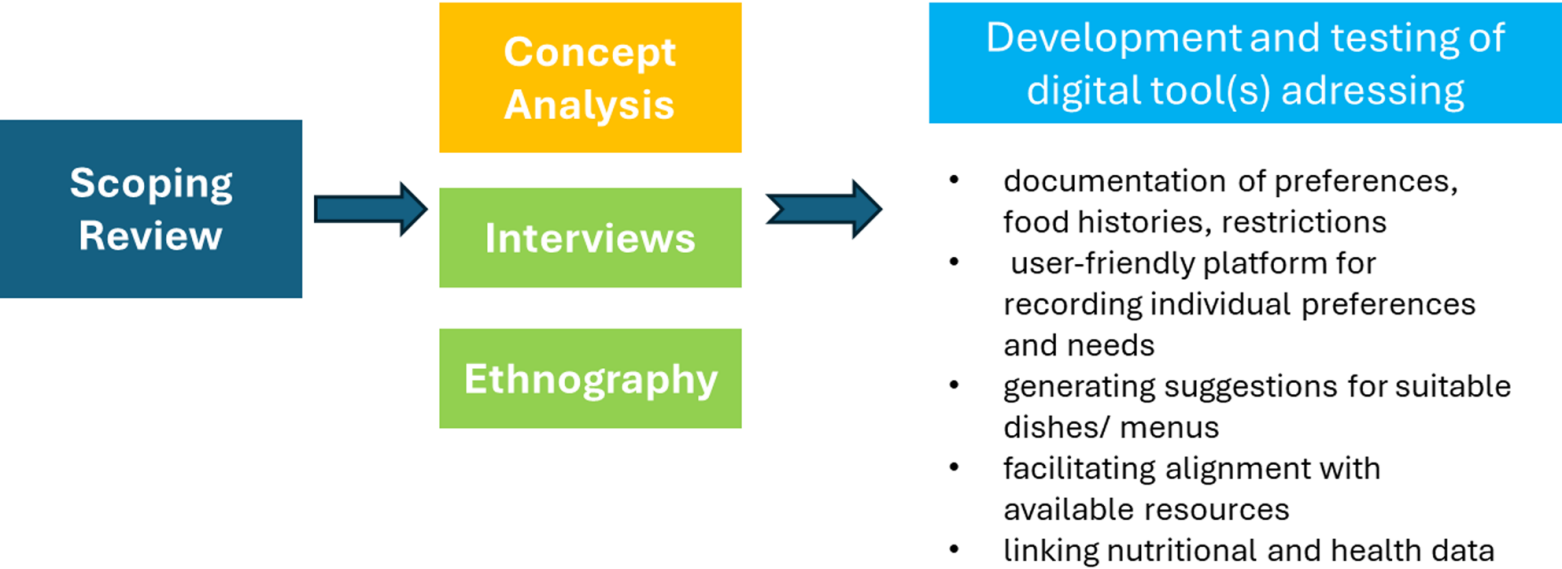
Conceptual model regarding the digital tool(s)

These findings influence the ongoing preparation regarding the development of a digital support system, but this needs, besides an further concept analysis, an empirical in-depth analysis through semi-structured interviews and participating observations in long-term care institutions (see Fig. 2):

It is essential to understand the cultural and religious eating habits, personal preferences, and health-related restrictions of residents, such as medication or illness, through dialogue. Although these conversations are time-consuming, they are crucial for promoting well-being, identity and sociality. In the first step, nursing staff therefore require support to systematically record and document preferences, food histories and any restrictions caused by illnesses or medications. Therefore, a digital system must provide a user-friendly platform for recording individual preferences and needs. Further functions could include generating suggestions for suitable dishes or menus and facilitating alignment with available resources. Smart documentation of food intake could also support nursing staff and help optimize personalized nutrition. Linking nutritional and health data can help identify risks such as malnutrition. However, developing the proposed functions requires a detailed analysis of the system’s requirements and its conceptualization based on the above-mentioned empirical research.

Only with a well-founded concept can such approaches be implemented in nursing homes and LTC facilities. It appears essential to conduct a comprehensive concept analysis to serve as a foundation for future research-driven developments in the field. Despite the critical importance of adequate nutrition and fluid intake, particularly for individuals requiring long-term care to prevent health deterioration, there remains a notable gap in the research literature regarding culturally and diversity-sensitive approaches to these essential needs. Given the demographic diversity present in many nations, it is imperative to acknowledge the pressing need for further investigation in this area.

## Limitations

This scoping review has several methodological limitations that warrant consideration. Notably, two of the included studies are integrative literature reviews that are restricted to English-language publications and fail to differentiate between ethno-specific and mainstream institutions [31]. Additionally, the study conducted by Harstäde et al. [33] exclusively examined literature from Western countries. The qualitative nature of the studies by Xiao et al. [32], Hannssen/Kuven [34], Chee [35], and Girard/El Mabchour [36] introduces further limitations; in particular, Girard/El Mabchour [36] employed ethnographic methods alongside qualitative interviews (for details see Table 2). Nonetheless, it provides valuable insights and highlights the critical need for further research.

## Conclusions

To date, no systematically developed, diversity- and culturally sensitive framework for managing residents’ nutrition and fluid intake in long-term care (LTC) facilities has been established. Findings from the NUTRI-SENSE scoping review provide preliminary indicators that may guide the nursing process and specify research directions for subsequent research and intervention development. In the context of international population aging and increasing LTC needs, the development and evaluation of culturally and diversity-sensitive nutrition and fluid intake strategies are of broad, cross-national relevance.

## Supplementary Information

Supplementary Material 1

## Data Availability

The datasets used and/or analyzed during the current study are available from the corresponding author on reasonable request. Please note: all datasets are in German.

## Abbreviations

CALD: Culturally and linguistically diverse
